# (3-Amino­pyrazin-4-ium-2-carboxyl­ate-κ^2^ *N* ^1^,*O*)diaqua­zinc(II) dinitrate

**DOI:** 10.1107/S1600536810042509

**Published:** 2010-10-30

**Authors:** Shan Gao, Seik Weng Ng

**Affiliations:** a College of Chemistry and Materials Science, Heilongjiang University, Harbin 150080, People’s Republic of China; b Department of Chemistry, University of Malaya, 50603 Kuala Lumpur, Malaysia

## Abstract

The water-coordinated Zn^II^ atom in the title salt, [Zn(C_5_H_5_N_3_O_2_)_2_(H_2_O)_2_](NO_3_)_2_, is *N*,*O*-chelated by a zwitterionic amino­pyraziniocarboxyl­ate unit; the metal atom, which lies on a center of inversion, shows an octa­hedral coordination. The nitrate ion inter­acts indirectly, through N—H⋯O hydrogen bonds. In the crystal, adjacent cations and anions are connected by O—H⋯O hydrogen bonds into a three-dimensional network motif. The crystal studied was a non-merohedral twin with two minor components of 15.1 (1) and 8.0 (1)%.

## Related literature

For a related structure, see: Tayebee *et al.* (2008 ▶). For the treatment of non-merohedral twins, see: Spek (2003 ▶).
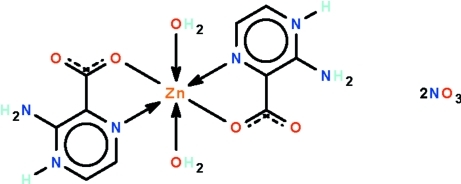


## Experimental

### 

#### Crystal data

[Zn(C_5_H_5_N_3_O_2_)_2_(H_2_O)_2_](NO_3_)_2_
*M* *_r_* = 503.66
Monoclinic, 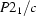

*a* = 13.4676 (14) Å
*b* = 9.7059 (9) Å
*c* = 6.6682 (6) Å
β = 96.610 (3)°
*V* = 865.84 (14) Å^3^
*Z* = 2
Mo *K*α radiation
μ = 1.51 mm^−1^
*T* = 293 K
0.24 × 0.21 × 0.18 mm

#### Data collection

Rigaku R-AXIS RAPID diffractometer
Absorption correction: multi-scan (*ABSCOR*; Higashi, 1995 ▶) *T*_min_ = 0.580, *T*_max_ = 1.000
8164 measured reflections
1983 independent reflections
1739 reflections with *I* > 2σ(*I*)
*R*_int_ = 0.053

#### Refinement

*R*[*F* ^2^ > 2σ(*F* ^2^)] = 0.060
*wR*(*F* ^2^) = 0.175
*S* = 1.15
1983 reflections
145 parameters
H-atom parameters constrained
Δρ_max_ = 1.37 e Å^−3^
Δρ_min_ = −1.66 e Å^−3^

### 

Data collection: *RAPID-AUTO* (Rigaku, 1998 ▶); cell refinement: *RAPID-AUTO*; data reduction: *CrystalStructure* (Rigaku/MSC, 2002 ▶); program(s) used to solve structure: *SHELXS97* (Sheldrick, 2008 ▶); program(s) used to refine structure: *SHELXL97* (Sheldrick, 2008 ▶); molecular graphics: *X-SEED* (Barbour, 2001 ▶); software used to prepare material for publication: *publCIF* (Westrip, 2010 ▶).

## Supplementary Material

Crystal structure: contains datablocks global, I. DOI: 10.1107/S1600536810042509/nk2064sup1.cif

Structure factors: contains datablocks I. DOI: 10.1107/S1600536810042509/nk2064Isup2.hkl

Additional supplementary materials:  crystallographic information; 3D view; checkCIF report

## Figures and Tables

**Table 1:** Hydrogen-bond geometry (Å, °)

*D*—H⋯*A*	*D*—H	H⋯*A*	*D*⋯*A*	*D*—H⋯*A*
O1w—H1w1⋯O1^i^	0.82	2.17	2.895 (5)	148
O1w—H1w2⋯O2^ii^	0.82	1.99	2.752 (6)	154
N2—H2⋯O3	0.88	1.86	2.703 (6)	161
N3—H31⋯O4	0.88	2.14	2.981 (6)	161
N3—H32⋯O5^iii^	0.88	2.33	2.994 (7)	133

Symmetry codes: (i) 
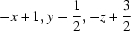
; (ii) 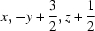; (iii) 
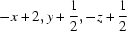
.

**Fig. 1.**
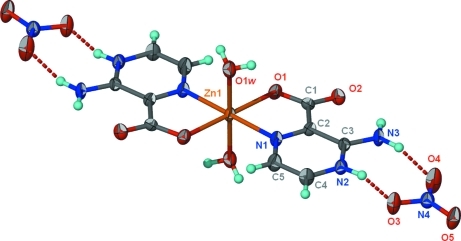
Displacement ellipsoid plot of Zn(H2O)2(C5H5N3O2)2 2NO3 at the 50% probability level; hydrogen atoms are drawn as spheres of arbitrary radius. Unlabeled atoms are related to labeled atoms by 1 - x, 1 - y, 1 - z.

## supplementary crystallographic information

### Comment

3-Aminopyrazine-2-carboxylic acid forms a number of aqua complexes with divalent transition metals in which the metal atom is *N*,*O*-chelated by the monoanion. In an attempt at the solution synthesis of the zinc derivative, the sodium ion used as a reactant is incorporated into the crystal structure (Tayebee *et al.*, 2008). In the present study, the attempt by a hydrothermal route yielded Zn(H_2_O)_2_(C_5_H_5_N_3_O_2_)_2_ 2NO_3_ (Scheme I, Fig. 1). The water-coordinated zinc atom in the salt is *N*,*O*-chelated by a zwitterionic aminopyraziniocarboxylate unit; the metal atom, which lies on a center of inversion, shows octahedral coordination. The nitrate ion interacts indirectly, through N–H···O hydrogen bonds. Adjacent cations and anions are connected by O–H···O hydrogen bonds into a three-dimensional network motif.

### Experimental

Zinc nitrate hexahydrate (0.30 g, 1 mmol), 3-aminopyrazine-2-carboxylic acid (0.28 g, 2 mmol), and sodium hydroxide (0.08 g 2 mmol) were dissolved in a H_2_O/DMF (12 ml, *v*/*v* = 2:1) solution. The mixture was sealed in a 25- ml Teflon-lined stainless steel bomb and held at 443 K for 3 d. The bomb was gradually cooled to room temperature, and yellow crystals were obtained after several days.

### Refinement

Hydrogen atoms were placed in calculated positions (C–H 0.93, N–H 0.88, O–H 0.82 Å) and were included in the refinement in the riding model approximation, with *U*(H) set to 1.2–1.5*U*(C,*N*,*O*). The crystal is a non-merohedral twin, with two minor components of 15.1 (1)% and 8.0 (1)%; *PLATON* (Spek, 2003) was used to separate the diffraction intensities into three domains. The final difference Fourier map had a peak at 0.92 Å from Zn1 and a hole at 0.98 Å from H1w2.

### Crystal data

**Table:**

[Zn(C_5_H_5_N_3_O_2_)_2_(H_2_O)_2_](NO_3_)_2_	*F*(000) = 512
*M**_r_* = 503.66	*D*_x_ = 1.932 Mg m^−^^3^
Monoclinic, *P*2_1_/*c*	Mo *K*α radiation, λ = 0.71073 Å
Hall symbol: -P 2ybc	Cell parameters from 7197 reflections
*a* = 13.4676 (14) Å	θ = 3.1–27.5°
*b* = 9.7059 (9) Å	µ = 1.51 mm^−^^1^
*c* = 6.6682 (6) Å	*T* = 293 K
β = 96.610 (3)°	Prism, yellow
*V* = 865.84 (14) Å^3^	0.24 × 0.21 × 0.18 mm
*Z* = 2	

### Data collection

**Table:**

Rigaku R-AXIS RAPID diffractometer	1983 independent reflections
Radiation source: fine-focus sealed tube	1739 reflections with *I* > 2σ(*I*)
graphite	*R*_int_ = 0.053
Detector resolution: 10.000 pixels mm^-1^	θ_max_ = 27.5°, θ_min_ = 3.1°
ω scans	*h* = −17→17
Absorption correction: multi-scan (*ABSCOR*; Higashi, 1995)	*k* = −11→12
*T*_min_ = 0.580, *T*_max_ = 1.000	*l* = −8→8
8164 measured reflections	

### Refinement

**Table:**

Refinement on *F*^2^	Primary atom site location: structure-invariant direct methods
Least-squares matrix: full	Secondary atom site location: difference Fourier map
*R*[*F*^2^ > 2σ(*F*^2^)] = 0.060	Hydrogen site location: inferred from neighbouring sites
*wR*(*F*^2^) = 0.175	H-atom parameters constrained
*S* = 1.15	*w* = 1/[σ^2^(*F*_o_^2^) + (0.0553*P*)^2^ + 4.4842*P*] where *P* = (*F*_o_^2^ + 2*F*_c_^2^)/3
1983 reflections	(Δ/σ)_max_ = 0.001
145 parameters	Δρ_max_ = 1.37 e Å^−^^3^
0 restraints	Δρ_min_ = −1.66 e Å^−^^3^

### Fractional atomic coordinates and isotropic or equivalent isotropic displacement parameters (Å^2^)

**Table:**

	*x*	*y*	*z*	*U*_iso_*/*U*_eq_	
Zn1	0.5000	0.5000	0.5000	0.0239 (3)	
O1	0.5443 (3)	0.7058 (4)	0.5390 (6)	0.0286 (8)	
O2	0.6686 (3)	0.8494 (4)	0.4874 (6)	0.0302 (8)	
O3	1.0229 (3)	0.4552 (5)	0.2346 (9)	0.0472 (12)	
O4	1.0421 (4)	0.6731 (5)	0.2591 (11)	0.0630 (17)	
O5	1.1600 (3)	0.5475 (5)	0.1631 (8)	0.0452 (11)	
O1W	0.5552 (3)	0.4400 (5)	0.8042 (6)	0.0388 (10)	
H1W1	0.5111	0.3991	0.8552	0.058*	
H1W2	0.5717	0.5089	0.8710	0.058*	
N1	0.6477 (3)	0.4895 (4)	0.4259 (7)	0.0280 (10)	
N2	0.8377 (3)	0.5065 (5)	0.3357 (8)	0.0288 (9)	
H2	0.9001	0.5100	0.3091	0.035*	
N3	0.8428 (3)	0.7410 (5)	0.3867 (8)	0.0331 (11)	
H31	0.9044	0.7417	0.3544	0.040*	
H32	0.8146	0.8183	0.4193	0.040*	
N4	1.0755 (3)	0.5598 (5)	0.2176 (8)	0.0320 (10)	
C1	0.6305 (4)	0.7331 (5)	0.4932 (8)	0.0235 (10)	
C2	0.6923 (4)	0.6113 (5)	0.4370 (7)	0.0221 (9)	
C3	0.7931 (4)	0.6243 (5)	0.3862 (8)	0.0245 (10)	
C4	0.7906 (4)	0.3840 (6)	0.3245 (10)	0.0357 (12)	
H4	0.8231	0.3057	0.2849	0.043*	
C5	0.6951 (4)	0.3757 (6)	0.3717 (10)	0.0358 (13)	
H5	0.6625	0.2910	0.3664	0.043*	

### Atomic displacement parameters (Å^2^)

**Table:**

	*U*^11^	*U*^22^	*U*^33^	*U*^12^	*U*^13^	*U*^23^
Zn1	0.0173 (4)	0.0251 (4)	0.0306 (5)	−0.0034 (3)	0.0077 (3)	0.0000 (3)
O1	0.0223 (17)	0.0259 (17)	0.039 (2)	0.0009 (14)	0.0117 (15)	−0.0037 (16)
O2	0.0281 (18)	0.0218 (17)	0.041 (2)	0.0008 (14)	0.0031 (16)	−0.0001 (15)
O3	0.031 (2)	0.026 (2)	0.089 (4)	−0.0043 (18)	0.024 (2)	−0.003 (2)
O4	0.048 (3)	0.025 (2)	0.124 (5)	0.001 (2)	0.045 (3)	−0.010 (3)
O5	0.023 (2)	0.040 (2)	0.075 (3)	−0.0003 (17)	0.020 (2)	−0.003 (2)
O1W	0.042 (2)	0.041 (2)	0.033 (2)	−0.0187 (19)	0.0033 (18)	0.0027 (18)
N1	0.023 (2)	0.024 (2)	0.039 (3)	−0.0002 (16)	0.0121 (18)	0.0015 (18)
N2	0.0153 (18)	0.037 (2)	0.035 (2)	0.0016 (17)	0.0058 (17)	0.0005 (19)
N3	0.023 (2)	0.031 (2)	0.046 (3)	−0.0061 (18)	0.011 (2)	0.000 (2)
N4	0.027 (2)	0.027 (2)	0.043 (3)	0.0000 (18)	0.0092 (19)	0.000 (2)
C1	0.022 (2)	0.025 (2)	0.023 (2)	0.0004 (18)	0.0022 (18)	−0.0004 (19)
C2	0.021 (2)	0.022 (2)	0.023 (2)	0.0012 (18)	0.0043 (18)	0.0022 (18)
C3	0.020 (2)	0.030 (3)	0.024 (2)	−0.0011 (18)	0.0035 (18)	0.000 (2)
C4	0.032 (3)	0.027 (3)	0.049 (3)	0.006 (2)	0.011 (3)	−0.003 (3)
C5	0.036 (3)	0.021 (2)	0.052 (4)	−0.001 (2)	0.013 (3)	−0.001 (2)

### Geometric parameters (Å, °)

**Table:**

Zn1—O1	2.092 (4)	N1—C2	1.324 (6)
Zn1—O1^i^	2.092 (4)	N1—C5	1.346 (7)
Zn1—N1^i^	2.107 (4)	N2—C4	1.345 (7)
Zn1—N1	2.107 (4)	N2—C3	1.353 (7)
Zn1—O1w^i^	2.158 (4)	N2—H2	0.8800
Zn1—O1w	2.158 (4)	N3—C3	1.315 (7)
O1—C1	1.262 (6)	N3—H31	0.8800
O2—C1	1.242 (6)	N3—H32	0.8800
O3—N4	1.250 (6)	C1—C2	1.517 (7)
O4—N4	1.232 (7)	C2—C3	1.442 (7)
O5—N4	1.240 (6)	C4—C5	1.362 (8)
O1W—H1W1	0.8200	C4—H4	0.9300
O1W—H1W2	0.8200	C5—H5	0.9300
			
O1—Zn1—O1^i^	180.0	C4—N2—H2	118.6
O1—Zn1—N1^i^	100.84 (15)	C3—N2—H2	118.6
O1^i^—Zn1—N1^i^	79.16 (15)	C3—N3—H31	120.0
O1—Zn1—N1	79.16 (15)	C3—N3—H32	120.0
O1^i^—Zn1—N1	100.84 (15)	H31—N3—H32	120.0
N1^i^—Zn1—N1	180.0	O4—N4—O5	121.5 (5)
O1—Zn1—O1W^i^	85.51 (16)	O4—N4—O3	118.6 (5)
O1^i^—Zn1—O1W^i^	94.49 (16)	O5—N4—O3	119.9 (5)
N1^i^—Zn1—O1W^i^	88.56 (18)	O2—C1—O1	126.4 (5)
N1—Zn1—O1W^i^	91.44 (18)	O2—C1—C2	117.4 (4)
O1—Zn1—O1W	94.49 (16)	O1—C1—C2	116.2 (4)
O1^i^—Zn1—O1W	85.51 (16)	N1—C2—C3	119.9 (4)
N1^i^—Zn1—O1W	91.44 (18)	N1—C2—C1	116.8 (4)
N1—Zn1—O1W	88.56 (18)	C3—C2—C1	123.2 (4)
O1W^i^—Zn1—O1W	180.0	N3—C3—N2	119.2 (4)
C1—O1—Zn1	115.3 (3)	N3—C3—C2	124.6 (5)
Zn1—O1W—H1W1	109.5	N2—C3—C2	116.2 (5)
Zn1—O1W—H1W2	109.5	N2—C4—C5	119.4 (5)
H1W1—O1W—H1W2	109.5	N2—C4—H4	120.3
C2—N1—C5	121.5 (5)	C5—C4—H4	120.3
C2—N1—Zn1	112.2 (3)	N1—C5—C4	120.2 (5)
C5—N1—Zn1	126.3 (4)	N1—C5—H5	119.9
C4—N2—C3	122.8 (4)	C4—C5—H5	119.9
			
N1^i^—Zn1—O1—C1	−176.2 (4)	C5—N1—C2—C1	178.1 (5)
N1—Zn1—O1—C1	3.8 (4)	Zn1—N1—C2—C1	−1.6 (6)
O1W^i^—Zn1—O1—C1	−88.5 (4)	O2—C1—C2—N1	−174.0 (5)
O1W—Zn1—O1—C1	91.5 (4)	O1—C1—C2—N1	5.0 (7)
O1—Zn1—N1—C2	−0.9 (4)	O2—C1—C2—C3	2.6 (7)
O1^i^—Zn1—N1—C2	179.1 (4)	O1—C1—C2—C3	−178.3 (5)
O1W^i^—Zn1—N1—C2	84.2 (4)	C4—N2—C3—N3	−177.5 (6)
O1W—Zn1—N1—C2	−95.8 (4)	C4—N2—C3—C2	2.7 (8)
O1—Zn1—N1—C5	179.4 (5)	N1—C2—C3—N3	178.0 (5)
O1^i^—Zn1—N1—C5	−0.6 (5)	C1—C2—C3—N3	1.4 (8)
O1W^i^—Zn1—N1—C5	−95.5 (5)	N1—C2—C3—N2	−2.2 (7)
O1W—Zn1—N1—C5	84.5 (5)	C1—C2—C3—N2	−178.8 (5)
Zn1—O1—C1—O2	173.2 (4)	C3—N2—C4—C5	−2.2 (10)
Zn1—O1—C1—C2	−5.7 (6)	C2—N1—C5—C4	−0.7 (10)
C5—N1—C2—C3	1.3 (8)	Zn1—N1—C5—C4	179.0 (5)
Zn1—N1—C2—C3	−178.4 (4)	N2—C4—C5—N1	1.1 (10)

Symmetry codes: (i) −*x*+1, −*y*+1, −*z*+1.

### Hydrogen-bond geometry (Å, °)

**Table:**

*D*—H···*A*	*D*—H	H···*A*	*D*···*A*	*D*—H···*A*
O1w—H1w1···O1^ii^	0.82	2.17	2.895 (5)	148
O1w—H1w2···O2^iii^	0.82	1.99	2.752 (6)	154
N2—H2···O3	0.88	1.86	2.703 (6)	161
N3—H31···O4	0.88	2.14	2.981 (6)	161
N3—H32···O5^iv^	0.88	2.33	2.994 (7)	133

Symmetry codes: (ii) −*x*+1, *y*−1/2, −*z*+3/2; (iii) *x*, −*y*+3/2, *z*+1/2; (iv) −*x*+2, *y*+1/2, −*z*+1/2.
